# Maternal mortality in the Gaza strip: a look at causes and solutions

**DOI:** 10.1186/s12884-018-2037-1

**Published:** 2018-10-11

**Authors:** Bettina Bӧttcher, Nasser Abu-El-Noor, Belal Aldabbour, Fadel Naim Naim, Yousef Aljeesh

**Affiliations:** 1 Faculty of Medicine, Islamic University of Gaza, P. O. Box 108, Gaza strip, Gaza, Palestine; 2 Faculty of Nursing, Islamic University of Gaza, P. O. Box 108, Gaza Strip, Gaza, Palestine

**Keywords:** Gaza-strip, Maternal mortality, Quality improvement, Medical documentation, Patient safety, Clinical audit, Palestine

## Abstract

**Background:**

Maternal mortality is an important health indicator for the overall health of a population. This study assessed the causes and contributing factors to maternal mortality that occurred in the Gaza-Strip between July 2014 and June 2015.

**Methods:**

This is a retrospective study that used both quantitative and qualitative data. The data were collected from available medical records, investigation reports, death certificates, and field interviews with healthcare professionals as well as families.

**Results:**

A total of 18 maternal mortalities occurred in Gaza between 1st July 2014 and June 30th 2015. Age at time of death ranged from 18 to 44 years, with 44.4% occurring before the age of 35 years. About 22.2% were primiparous, while 55.6% were grand multiparous women. The most common causes of death were sepsis, postpartum haemorrhage, and pulmonary embolism.

The most striking deficiency was very poor medical documentation which was observed in 17 cases (94%). In addition, poor communication between doctors and women and their families or among healthcare teams was noticed in nine cases (50%). These were repeatedly described by families during interviews. Further aspects surfacing in many interviews were distrust by families towards clinicians and poor understanding of health conditions by women. Other factors included socioeconomic conditions, poor antenatal attendance and the impact of the 2014 war.

Low morale among medical staff was expressed by most interviewed clinicians, as well as the fear of being blamed by families and management in case of adverse events. Substandard care and lack of appropriate supervision were also found in some cases.

**Conclusions:**

This study revealed deficiencies in maternity care, some of which were linked to the socioeconomic situation and the 2014 war. Others show poor implementation of clinical guidelines and lack of professional skills in communication and teamwork. Specialised training should be offered for clinicians in order to improve these aspects. However, the most striking deficiency was the extremely poor documentation, reflecting a lack of awareness among clinicians regarding its importance. Local policymakers should focus on systematic application of quality improvement strategies in order to achieve greater patient safety and further reductions in the maternal mortality rate.

## Background

Maternal mortality is an important health indicator, widely acknowledged as a general indicator of the overall health of a population, the status of women in a society, and the functioning of the healthcare system. Maternal mortality is defined as death of a woman during pregnancy or within 42 days after childbirth, spontaneous abortion or termination of pregnancy, but excluding deaths from incidental or accidental causes [1]. In the year 2000, the United Nations set ten Millennium goals to be reached by 2015. Among these was Millennium Goal Number five: “To reduce maternal mortality by three quarters from 1990 to 2015” [2]. Many Middle Eastern countries are believed to have reached this goal. However, this was not achieved globally and conflicts in the Region have made it more difficult to reach this goal at a regional level [3].

According to the Palestinian Ministry of Health (MoH), the Maternal Mortality Rate (MMR) in the Gaza-Strip was 30/100,000 live births in 2014, and 25 in 2015 [4, 5], which differed from that in the West Bank with 20/100, 000 in 2014 [4]. On the other hand, MMR was 7 in Israel, 58 in Jordan and 33 in Egypt [3, 5, 6]. The Gaza-Strip suffers from several severe social and economic challenges due to a blockade that has been imposed on Gaza since 2006, which might have an impact on health indicators including maternal mortality [7]. This may explain the obvious difference in the MMR between the Gaza-Strip and the West Bank (the two parts of Palestine). Moreover, Abdo et al. suggested that recurring conflicts affecting the Gaza-Strip also contributed to differing maternal mortality rates [8].

This study aimed to identify causes of maternal mortality in the Gaza-Strip that occurred between 1st July 2014 and 30th June 2015. Furthermore, this study sought to explore the relationship between MMR and socioeconomic status as well as the relationship between MMR and access to healthcare facilities during the war that took place in July and August 2014. Finally, it was intended to identify ways of improving the quality of maternal health services and reduce MMR in the Gaza-Strip to achieve the WHO goal of reduction in MMR by two-thirds [2].

## Methods

A descriptive, retrospective design that included a mixed (triangulation) approach of quantitative and qualitative data collection was used in this study. The use of both quantitative and qualitative approaches strengthens the design and reduces any weaknesses in either approach [9, 10]. It also provides richer and more in-depth data that will reduce bias of using a single method [11, 12].

The study population included all women who died during pregnancy or within 42 days post-delivery or abortion that occurred between 1st July 2014 and 30th June 2015 in the Gaza-Strip. A central register of maternal deaths exists in the Gaza-Strip and is kept by the Palestinian Ministry of Health. Identification and registration of these cases is mandatory for all hospitals. During the study period, 18 maternal deaths, that met the definition of maternal mortality, had been registered and were included in this study [1]. Pregnant women who died due to accidents were excluded from the study, as they do not meet the WHO definition of maternal mortality [1]. The available clinical notes, investigation reports and death certificates for the 18 women were reviewed. Data collection sheets, developed by the research team, were completed for each case. The sheet covered five main domains which included: socioeconomic status, past medical history, obstetrical history, received antenatal care, and the index pregnancy.

Interviews were conducted with the families of the deceased women. When possible, home visits were made to meet these families. In 14 cases the husbands were interviewed, while four of the interviews involved other family members in addition to the husband, and in two cases only relatives without the husband of the deceased were interviewed. The research team could not interview the family members of two women; one family refused any contact with the research team and for the other family, contact information could not be found. In seven cases, for the convenience of family members, the interviews were made by phone. Besides that, the research team members interviewed 12 medical clinicians who had been involved in caring for the deceased women. In total, three medical directors were interviewed, eight consultants, including four obstetricians, two intensive care physicians, one cardiologist and one microbiologist, as well as one primary care manager. Interviews with clinicians lasted between 20 to 90 min, while interviews with family members took more time, ranging from 45 to 100 min.

During the data collection process, several obstacles and challenges had to be overcome. Firstly, finding the medical case notes for the deceased women; only 12 case notes could be located. In the remaining cases, notes were not available, mostly due to poor contact with the health services or care providers during the war in July and August of 2014. Similarly, contact information for women were not recorded systematically in the notes. Contact phone numbers were found to be scribbled at odd places in the case notes, but were completely missing in eight cases. Therefore, contact details were obtained informally in these cases, by asking people who knew the families of the deceased women.

### Ethical considerations

Ethical approval for this study was obtained from the Human Resources Department of the Palestinian Ministry of Health (MoH), which is the body in Gaza to issue ethical and administrative approvals for studies involving patients and their families. Further approval to conduct the study was obtained from the administrative bodies of the governmental and one private hospitals, that had provided care for the deceased women. Before conducting the interviews with family members or healthcare providers, the aims of the study were explained to the participants, and a formal consent to participate in this study was obtained. Prior to data collection and after obtaining ethical approval from the Human Resources Department, verbal consent was obtained from participants who preferred to be interviewed over the phone, while written consent was taken from those interviewed face-to-face. Participants were informed that they had the right to refuse participation in the study or to withdraw at any time without being penalized. The collected data were kept under high confidentiality and anonymity as each case was assigned a code number. The women’s confidentiality was preserved throughout the study.

### Data analysis

Data analysis was done with the help of EXCEL (Microsoft Inc.) and mainly in terms of descriptive statistics with means, standard deviations and percentages. For qualitative data analysis, the researchers read the transcripts of the interviews independently. Transcripts were reviewed several times by each researcher in order to gain an increased understanding of the study phenomenon at multiple levels if possible [12]. While reading the transcripts, each researcher coded data and organized the participants’ sentences and paragraphs of the interviews with similar properties into different themes and subthemes [11]. After each researcher finished his/her analysis, the research team met in person and discussed patterns relating to a core category. During the meeting, data were coded using constant comparison among themes from each researcher until a consensus was reached on the common core themes of the study. From these themes, the main categories of contributing factors were identified and quantified as listed in Table 1. The manuscripts and coding of the data was read by several experts to ensure rigor of the study and avoid bias [13, 14].

**Table 1:** Contributing Factors to Poor Outcome and Aspects of Care

Contributory Factor	Total Number	Percentage of Total
Substandard care and poor documentation	17	94.4%
Poor communication	9	50%
Impact of war	4	22.2%
Poor educational achievements of patients	3	16.7%
Socioeconomic factors and cultural beliefs	5	27.8%
Poor understanding of illness/ self-neglect	4	22.2%
Access difficulties	2	11.1%
Irregular/ late antenatal care attendance	5	27.8%

## Results

### Characteristics of deceased women

The age of the 18 women, who died during the study period, ranged from 18 to 44 years, with a mean age of 33.5 ± 7.1 years. From these, four women (22%) were primiparous, while ten (55%) were grand multiparous women (para five and more). The majority of deaths occurred during the antepartum period (10 cases; 55%) followed by the postpartum period (seven cases; 38.9%). Only one death occurred intrapartum. Eight women (44%) had been identified as high risk pregnancies due to a variety of reasons including previous Caesarean section, pregnancy-induced hypertension (complicated by gestational diabetes mellitus), epilepsy, poorly-controlled bronchial asthma, deep vein thrombosis during a previous pregnancy and one had an aortic stenosis.

### Causes and frequency of mortalities over time

**Fig. 1:**
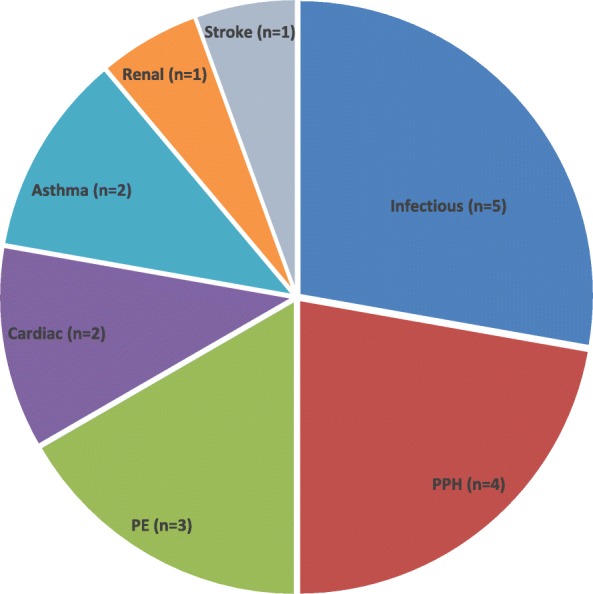
Causes of MMR by frequency: Infectious (*n* = 5), Postpartum haemorrhage (*n* = 4), Pulmonary embolism (*n* = 3), Cardiac (*n* = 2), Asthma (*n* = 2), Renal (*n* = 1), Stroke (*n* = 1)

**Fig. 2:**
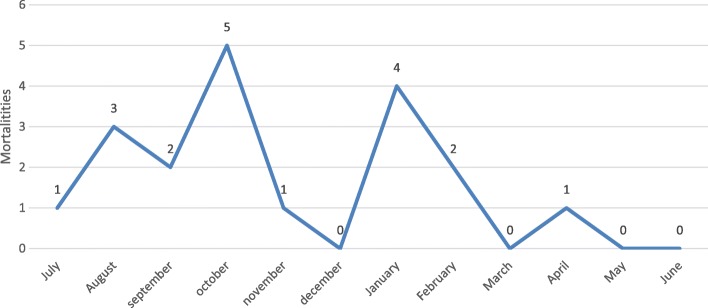
Frequencies of Mortalities per Month

The most frequent causes for mortality were found to be pulmonary embolism (PE), infection/septicemia, and post-partum hemorrhage (PPH) (Fig. 1). Four women (22.2%) died during the war in July and August 2014 (Fig. 2). A further two peaks were noted in October 2014, following the war, when five women (27.8%) died and then again in January 2015 with another four (22.2%) deaths, indicating exposure to cold weather due to poor home insulation as a possible factor, confirmed by home visits to families whose relative died of uncontrolled asthma.

### Contributing factors to mortalities

From the collected data, including the interviews, several recurring themes could be identified as contributing factors to these deaths, which were mainly poor documentation (including patient notes, progress reports, operation and birth records as well as investigation results), poor communication within teams and between healthcare professionals and women and their families, socioeconomic factors as well as irregular, late or no antenatal care attendance (Table 1). Furthermore, direct impact of the war as well as self-neglect contributed to some maternal deaths. Poor educational achievements were evident in three women (which included one woman who had not benefitted from the most basic schooling and two who had only completed four years of education) (Table 1).

#### Substandard care and poor documentation

Evidence of substandard care was found in the care of 10 women (55.6%). In all of these, documentation was significantly substandard, compounding the poor care. In particular, no attention was being paid to recording timings of events or the given medications in any of the medical case notes. Essential details, such as estimated blood loss in cases of haemorrhage or intraoperative complications in case of Caesarean sections, were also consistently missing in the notes. The condition of two women (11.1%), deteriorated slowly overnight, which was not recognised by healthcare providers. Lack of supervision was evident in the care of some women, as in the case of one woman who suffered from severe dyspnoea, a serious sign in a young adult, which was not recognised to have been caused by irritation of the diaphragm. As a consequence, the hypovolaemia from the massive intra-abdominal haemorrhage had been overseen by the resident doctor. Senior obstetric help and support were not called, despite ongoing symptoms.

#### Poor communication

Poor communication was evident in the care of nine women (50%) and included a lack of safety netting, inappropriate reassurance of women and their families, even if the women’s conditions were critical, poor skills at breaking bad news and avoiding to see families following poor outcomes. One husband said: “*They did a CT scan* (computed tomography) *and blood tests and the doctor told us that everything was 100% good and we went home.*” However, his wife had a severe and ongoing headache and died within 48 h due to meningitis, after staying at home and re-presenting very late to the hospital. At the time of discharge, she had been completely reassured that everything was OK and had not been instructed to return in case of ongoing or worsening symptoms, illustrating the close connection between poor communication and substandard care. In another case, the husband reported that he asked a doctor about the condition of his wife, who had been admitted to the intensive care unit (ICU), and was told that: *“She is fine. Everything is good*.” But his wife died within one hour after this reassurance was given to him. Another husband illustrated his experience in the following words:

*“My wife was unconscious on that day. Her condition was getting worse and worse every day… the ICU doctors did not give us accurate information about her condition. Even when they discovered that she had kidney failure, they told me that she will need kidney dialysis only one time and her kidney will come back to work.”*

The transfer process of two women between hospitals within the Gaza-Strip was impeded by poor communication between healthcare professionals, leading to an extreme delay in care provided to the women, probably contributing to their death. This was described by one husband as follows: “*In the last week of her pregnancy, we went to Hospital B, but they referred us to Hospital A. When we arrived there, they refused to receive us. So, we went back to Hospital B, who sent us back to [*Hospital A*]* (a more specialized hospital)*, who refused to receive my wife again. This happened four times in the midst of the war.”* Further factors contributing to this delay included a lack of agreed referral protocols for the transfer of patients and a situation of extreme stress also for medical personnel during the war. A formal referral and transfer policy has since been agreed and implemented. One director said: “*These were difficult conditions and lack of agreement on the transfer process contributed to the delays during the war. We have since come together and agreed on a transfer policy*”.

Poor communication between healthcare professionals and its impact on the care delivered to women was also described by physicians. One physician recounted the care of a patient with severe aortic stenosis, where obstetricians and cardiologists could not establish effective multidisciplinary care. The woman was cared for on the cardiac care unit for a large part of her pregnancy. The physician related: *“The obstetric team did not visit her regularly, but only when they were called to see the woman. No care arrangements were made by obstetric and cardiac teams together.”*

#### Impact of war

The clinical care of four women (22.2%) was directly impacted by the ongoing war in July and August 2014, mainly with limitations of safe transportation to access care or impact of smoke from explosions, as one husband reported: “*They* (the doctors) *told me that the difficulty of breathing was due to the smoke from the explosions of rockets.*” Another two families found that certain stressors had an effect on patients even after hostilities had ceased. At least seven families (38.9%) were displaced during the war and found shelter either in a mosque, a school or with relatives. However, only six deaths (33.3%) occurred during or in the first month following the war (Fig. 2).

#### Socioeconomic factors

Socioeconomic factors were immediate contributors to the poor outcome in the care of five women (27.8%). Two women with bronchial asthma died in January, the coldest month of the year, and lived in inadequate, very cold and poorly insulated, housing (Fig. 2).

#### Cultural beliefs

Social pressure for having male children contributed to a pregnancy against medical advice and a concealed pregnancy without antenatal care in two women, who were known to have cardiovascular disease, one with aortic stenosis and one with hypertension. One husband expressed his unawareness of his wife’s pregnancy as follows: “*She had told me that she had a coil. But after her death, I learned from her sister that it had been removed as she wanted another son.*” The family of the woman with aortic stenosis described that she had met another woman “*with a heart problem like hers*,” who had had three children and believed this was also possible for her. She already had one daughter and had been strongly advised against a further pregnancy, but her maternal aunt said: “*She believed she could have more children as the other woman she had met and she wanted a son.*”

### Distrust in physicians

Eight of the interviewed families (44.4%) expressed a high degree of disappointment and the view that their relatives had been treated negligently. In five cases (28%), this view was very strong. To express his distrust in physicians, one husband described them as *“licensed killers”.* In all of these cases, there were evident communication shortfalls between medical staff and families. Eight cases (44.4%) reported a lack of information given to them or ‘inappropriate reassurance’, meaning that husbands had been reassured that everything was OK regarding the condition of their wives until shortly before their deaths.

### Low morale among clinicians

When talking to clinicians, low morale was found in most cases. This was largely due to high workloads and pressures that were rewarded with only small salaries, which were not received regularly. Employees in the Palestinian Ministry of Health receive only 50% of their monthly salary every 40 days for the last few years, including the study period. Furthermore, doctors perceived a lack of support from managers and the MoH in case of adverse events. On the contrary, the health officials and managers were perceived by clinicians to seek individuals to blame and discipline for such events. As a result, clinicians avoided involvement in difficult cases, as in the case of a 20-year old woman with aortic stenosis. She had been warned against pregnancy and was told that this might endanger her life. In spite of that, she decided to get pregnant. However, she did not receive any antenatal care until she was in the second trimester when she had already developed dyspnoea. Although she was advised to terminate her pregnancy, at this stage, obstetricians avoided undertaking such a procedure, for fear of her death occurring in the process and being blamed for it. One obstetrician described this problem and said: “*If she died on the table, the doctors would be blamed by families and managers.*” This led to care being delivered reactively rather than proactively. Clinicians described being trapped in a culture of blame. One obstetrician described the team, who investigated maternal mortalities, as “*policemen*” who search for faults.

## Discussion

The causes of maternal death that were found in this study were close to the causes reported in a previous study (5). No major changes occurred in the frequency of PE and PPH as a cause of maternal death. However, an increase was noted in the percentage of women who died due to infectious causes, bronchial asthma and renal disease. On the other hand, a decrease occurred in the percentage of women dying due to cardiovascular diseases (Table 2). Hypertension was only implicated as a contributory factor to death in one case. This is probably an expression of the excellent routine antenatal care available to women across the Gaza-Strip free of charge, which was described in one study as high quality care [15].

**Table 2:** Frequency of Causes of Death in Percent in 2008/09 and 2014/15

Cause of Death	2008/09(16) *N* = 11 (2008) *N* = 18 (2009) *N* = 30 (total – 2 years)	2014/15 Current study *N* = 18 (1 year)
Pulmonary Embolism	20.0%	16.7%
Postpartum Haemorrhage	16.7%	22.2%
Infectious Causes	16.7%	27.8%
Cardiovascular Disease	30.0%	16.7%
Bronchial Asthma	0	11.1%
Renal Disease	0	5.6%
Others	16.7%	0

Determination of causes of maternal death was done without postmortem examination in all cases. In one case an evident discrepancy was noted between the ‘official’ cause of death ‘postpartum haemorrhage’ and the one supported by interviews with clinicians, who suspected ‘pulmonary embolism’. This difference in opinion between attending obstetricians and investigators, which could not be resolved, demonstrates the poor communication and lack of teamwork among clinicians, managers and investigation teams. Rather than focusing on learning points and improvements as well as system failures, adverse event investigators from the MoH were perceived by clinicians to be more concerned about pointing out wrongful acts and blame rather than investigating system failures.

The lack of interest in investigating and confirming causes of maternal death, once they had occurred, was evident. For example, in two cases of sepsis, samples had been obtained for investigation (one sample from a pleural infusion as well as blood cultures), but laboratory results were not followed up or documented in the notes. It appeared that clinicians felt that documentation was not necessary and did not feel the obligation to follow up on them after the woman had died.

In the Gaza-Strip, antenatal care is provided by governmental as well as UNRWA (United Nations Relief and Works Agency) clinics to all women free of charge and meets the basic care needs of all pregnant women [15]. In total, 95.3% of women in Gaza were reported to have taken advantage of this in 2015 [5]. However, ultrasound examinations are not routinely available, so women have to seek these in the private sector; a service that not all women can afford. In this study, 22.2% of the mortalities did not take advantage of regular antenatal care and this is a large proportion compared to the < 5% of women in the general population of Gaza, who are reported to have < 4 antenatal visits [5, 7].

The vast majority of women in the Gaza-Strip give birth in governmental hospitals, which was also the case in this sample. Only one woman in this sample had delivered in a private hospital, where she suffered a PPH and was transferred instantly to the local governmental hospital. Postnatal care is reported to reach 91.8% of women in the Gaza-Strip, attending a healthcare institution for a postnatal care visit within two days after childbirth [16].

A broad lack of trust by patients in the healthcare system of Gaza became evident, reinforced by families of deceased women feeling excluded by healthcare teams. Inappropriate reassurance by the medical teams, as well as avoidance of seeing the family shortly following their relative’s death, revealed a lack of skills by clinicians in breaking bad news. Yet, the lack of effective communication has been shown to be an obstacle to providing appropriate healthcare and safeguarding patient safety [17–19]. Effective communication between teams, amongst healthcare professionals and between healthcare professionals and their patients is essential in the transfer of information (handover), getting a clear picture of the problem, referral between hospitals, follow up and safety netting. To this effect, including the patient as part of the team is essential for obtaining information and decision making [18, 20, 21]. In this study, one or all of these aspects were missing or insufficient in 66% of the cases.

The extremely poor standard of documentation shown in this study has proven another obstacle in providing safe maternity care. Unfortunately, this has been shown to be a widespread problem in the Gaza-Strip [22, 23]. Meticulous, contemporaneous and accurate documentation is the backbone of evaluation and quality improvement in any healthcare system [24]. This study shows evidence of significantly substandard documentation even in the most severe and moribund cases. However, documentation is essential for patient care and safety as well as for effective clinical audit. The lack of simple measures of documentation, such as the use of early warning scores, contributed to maternal mortality in this study and underline documentation to be clinicians’ duty and not a choice [25, 26].

The development of a culture of openness, encouraging clinical audit and transparency, is not optional when improving the healthcare system. Clinicians often voiced fears of transparency in adverse events, suggesting that this would lead to less trust by patients in local clinicians, which has also been identified in other studies [27]. Clinical audit allows the identification of weaknesses and facilitates improvements. As such it is the cornerstone of quality improvement processes in healthcare and mandatory for ensuring patient safety [28, 29]. Contrary to current local opinion, it would increase trust in the healthcare system [30].

The impact of the 2014 war, as well as socioeconomic factors, were great and affected about 50% of women. Subtler and more difficult to measure is the influence of the blockade, which has been imposed on the Gaza-Strip since 2006. It has led not only to shortages and to varying availability of drugs, disposables, and medical equipment, but it also caused difficulties or impossibilities for healthcare professionals from Gaza to join international conferences and advanced medical training, contributing to increasing isolation of healthcare professionals in the Gaza-Strip [31]. Due to these factors, professionals in Gaza do not partake easily in global developments and advances in medicine and practice. This might be one factor contributing to the fact that clinical audit and quality improvement initiatives have largely been absent in Gaza and no significance is currently attached to adequate documentation in medical notes. The healthcare system seems to have ‘missed out’ on these important developments, despite access to the internet. Currently, local initiatives and debates are gathering speed, promoting clinical audit as well as local quality improvement initiatives to benefit the healthcare system across the Gaza-Strip. This has been supported by non-governmental organisations, local universities, visiting teams of clinicians, as well as, most recently official support by the Palestinian MoH and also the Palestinian Medical Council. These interventions have brought the process of continuous quality improvement in healthcare into focus, but have yet to reach all clinicians.

## Conclusion

Multiple factors were identified to be contributing to maternal mortality in the Gaza-Strip. In order to reduce MMR, in an attempt to meet the WHO goal, further measures, such as improvement of documentation, clinical audit activity and development of skills in communication are all relatively inexpensive interventions, which are within the reach of the healthcare system in the Gaza-Strip. It is due to local leadership to pick up this baton and realise greater patient safety and reduction in MMR by more systematically applying quality improvement strategies.

## Abbreviations

ICU: Intensive Care Unit
MMR: Maternal Mortality Rate
MoH: Palestinian Ministry of Health
NGO: Non-Governmental Organisation
PE: Pulmonary Embolism
PPH: Postpartum Haemorrhage
UNFPA: United Nations Population Fund
UNRWA: United Nations Relief and Works Agency
WHO: World Health Organisation
